# Bacteriology of Adenoids and Tonsils in Children With Recurrent Adenotonsillitis

**DOI:** 10.7759/cureus.47650

**Published:** 2023-10-25

**Authors:** Pulkit Mehrotra, L Somu

**Affiliations:** 1 Forensic Medicine, Sri Ramachandra Institute of Higher Education and Research, Chennai, IND; 2 College of Medicine, Sri Ramachandra Institute of Higher Education and Research, Chennai, IND; 3 Otolaryngology, Sri Ramachandra Medical College, Chennai, IND

**Keywords:** india, children, adenotonsillitis, adenoids, tonsils

## Abstract

Introduction: Recurrent adenotonsillitis (AT) commonly affects children and may be associated with various complications. Infections are common etiology, and microbial profiles may vary widely in different cases. In this study, we evaluated the bacterial profile and antibiotic sensitivity of pathogens identified in tonsil and adenoid core cultures in children with recurrent AT.

Methods: In this cross-sectional, observational study, culture and antibiotic sensitivity were performed from tonsil and adenoid core samples obtained after adenotonsillectomy of children (5 to 18 years) with recurrent AT. Children who had received antibiotics within one week before surgery were excluded. Drug sensitivity was performed only for drugs available on the hospital panel list.

Results: Bacterial growth was observed in 83 (91.2%) tonsil core cultures (n=91) and 43 (79.6%) adenoid core cultures (n=54). In the tonsil and adenoid core cultures, poly-microbial growth was seen in 25 (27.0%) and 11 (25.6%) children, respectively. From the tonsil core cultures, the majority of the bacteria were sensitive to ciprofloxacin, ampicillin, piperacillin-tazobactam, cefoperazone-sulbactam, ceftazidime, cefotaxime, levofloxacin. From the adenoid core culture, the majority of the bacteria were sensitive to ciprofloxacin, ampicillin, piperacillin-tazobactam, cefoperazone-sulbactam, cephalexin, and cefotaxime.

Conclusion: In recurrent AT, polymicrobial growth is not uncommon in both tonsil and adenoid core cultures. Identifying the correct pathogens and their antibiotic sensitivity patterns can help plan treatment strategies for the effective management of recurrent AT.

## Introduction

Globally, upper respiratory infection (URI) is a commonly encountered illness with the highest incidence in children below the age of five years [1]. Recurrent adenotonsillitis (AT) can lead to frequent rhinosinusitis, breathing difficulties, chronic ear infections, disturbed sleep, and mood changes [2-4]. Adenotonsillectomy is the ultimate treatment for recurrent AT. Operative intervention rates are significantly higher in children than in adolescents, and it becomes essential in children with recurrent AT and obstructive sleep apnea [5,6]. Pathogenic bacterial infections are the most common cause of AT, but microbiology may vary substantially in recurrent cases. Mixed aerobic-anaerobic polymicrobial infections could underlie recurrent AT cases [7]. In such cases, a high bacterial load may lead to bacterial deposition in the tonsillar and adenoid core. Chronic persistent infections may also lead to the formation of biofilms [8]. Studies have reported the isolation of the anaerobes and resistant pathogens from the tonsillar and adenoid cultures [9,10]. In India, a substantial proportion of children suffer from recurrent AT. Studies have shown significant variability in pathogens isolated from such children. Polymicrobial infections, as well as resistant pathogens, are commonly observed [11,12]. Identification of the causative pathogen can help plan the treatment strategies and selection of antibiotics. This study explored the microbiological profile and antibiotic sensitivity pattern of pathogens identified from adenoid and tonsil core cultures in children suffering from recurrent AT.

## Materials and methods

Design and setting

This was a cross-sectional, observational study. The study was conducted at the Department of Otorhinolaryngology (ENT) of Sri Ramachandra Institute of Higher Education and Research, Chennai, India. Approval of the study protocol (Protocol no. CSP/19/JUN/78/257) was granted by our Institutional Ethics Committee at the Sri Ramchandra Institute of Higher Education and Research, Chennai. Parents of all children participating in the trial provided informed written consent before initiating the study.

Population

Children with recurrent adenotonsillitis were screened for inclusion in the study. All participants were enrolled from October 2019 to October 2021. Children aged between 5 to 18 years diagnosed with recurrent adenotonsillitis who underwent adenotonsillectomy with no antibiotic coverage one week before the surgery were included in the study. Recurrent adeno-tonsillitis was defined as seven or more attacks of tonsillitis within the past one year, five or more attacks per year within two years, or three or more attacks per year within the past three years [13].

Procedures

After pre-anesthetic workup, children with recurrent AT were subjected to adenotonsillectomy under general anesthesia. The child was placed under general anesthesia with orotracheal intubation in the Rose position. Mouth opened using Boyle Davis mouth gag and tongue blade of appropriate size and fixed using Draffin bipod stand. The adenoids were curetted out using a St Clair Thompson adenoid curette (Surrey, England) till an adequate airway was achieved. The palatine tonsils were dissected and snared out. The tonsil and adenoid tissues were removed. The tonsil and adenoids were dissected in half, with the core tissue being taken and collected in a sterile culture tube. The core specimen was transported after half to one hour, processed, and later inoculated into blood, chocolate, and MacConkey’s agar plates. Bacterial identification was performed as per conventional procedures. Based on the preliminary growth, antibiotic specificity was checked for a panel of antibiotics available on the hospital protocol.

Statistical analysis

Data from case records were organized and entered into a Microsoft spreadsheet (Redmond, USA) and analyzed with the same. Categorical variables were presented with frequency and percentages.

## Results

A total of 91 patients who fit the inclusion criteria were included in the study. Throat swabs were taken for them and sent for culture and sensitivity. Table 1 describes the culture prevalence in throat swabs, tonsils, and adenoid cultures. Out of 91, 83 (91.2%) tonsil core samples showed the growth of microorganisms. From 54 adenoid cultures, 43 (79.6%) showed growth. None of the throat swabs showed any growth. Poly-microbial growth was seen in 25 (30.0%) tonsil core cultures and 11 (25.6%) adenoid cultures.

**Table 1:** Culture positivity rate in throat swab, tonsil, and adenoid culture.

Specimen	n (%)
Culture growth	126/145 (86.9)
Tonsil	83/91 (91.2)
Adenoid	43/54 (79.6)
Type of growth	
Tonsil (n=83)	
Mono-microbial	58 (69.9)
Poly-microbial	25 (30.1)
Adenoid (n=54)	
Mono-microbial	32 (74.4)
Poly-microbial	11 (25.6)

The microbial profile of the organisms isolated is shown in Table 2. From a total of 83 tonsil core cultures, gram-positive bacteria growth was found in 62 (74.7%), while gram-negative bacteria growth was seen in 44 (53.0%) cultures. In the adenoid core culture, the gram-positive bacteria growth dominated (39 [90.7%]). Among Gram-positive bacteria, the growth of *Streptococcus* species, *Staphylococcus aureus*, *Enterococcus faecalis*, *Enterococcus faecium*, and *Streptococcus pyogenes* were seen in 28.9%, 18.1%, 12.0%, 10.8% and 4.8% of tonsil core cultures, respectively, whereas it was seen in 25.6%, 20.9%, 18.6%, 16.3% and 0% of the adenoid core cultures, respectively. Similarly, in the Gram-negative bacteria, the growth of *Enterobacter* species, *Klebsiella pneumonia*, and *Pseudomonas aeruginosa* was present in 34.0%, 29.5%, and 22.7% of tonsil core cultures and 0%, 20.9%, and 14.0% of adenoid core cultures, respectively.

**Table 2:** Microbial profile of organisms isolated in tonsil and adenoid culture.

Microbiological profile	Tonsil (n=83)	Adenoid (n=43)
Gram positive	62 (74.7)	39 (90.7)
Streptococcus species	24 (28.9)	11 (25.6)
Staphylococcus aureus	15 (18.1)	9 (20.9)
Enterococcus faecalis	10 (12.0)	8 (18.6)
Enterococcus faecium	9 (10.8)	7 (16.3)
Streptococcus pyogenes	4 (4.8)	0
Coagulase negative Staphylococcus	0	4 (9.3)
Gram negative	44 (53.0)	15 (34.9)
*Enterobacter* species	15 (34)	0
Klebsiella pneumoniae	13 (29.5)	9 (20.9)
Pseudomonas aeruginosa	10 (22.7)	6 (14.0)
*Acinetobacter* species	2 (2.4)	0
Escherichia coli	2 (2.4)	0
Enterobacter cloacae	2 (2.4)	0

Tables 3, 4 depict the antibiotic sensitivity pattern in the tonsil core and adenoid core cultures. *Enterobacter spp.* the tonsil core culture showed sensitivity to amikacin, cefoperazone-sulbactam, ciprofloxacin, and piperacillin-tazobactam. A similar sensitivity pattern was seen with *K. pneumoniae* as well as *P. aeruginosa*. The *Streptococcus spp*. showed good sensitivity to ampicillin, amoxicillin-clavulanic acid, and certain cephalosporins such as cefotaxime. Cephalosporins had good activity against *S. aureus*. Among the isolates from the adenoid core culture, *K. pneumoniae* showed good sensitivity to amikacin mainly and piperacillin-tazobactam to some extent. The majority of tested antibiotics did not show good sensitivity to *P. aeruginosa* isolated from adenoid core culture, and the same was true for *Streptococcus spp.* and *S. aureus* as well.

**Table 3:** Antibiotic sensitivity pattern in bacteria isolated from tonsil core culture. Data presented as n (%) for pathogen sensitivity to a specific antibiotic

Drug	*Enterobacter spp.* (n=15)	*K. pneumoniae* (n=13)	*P. aeruginosa* (n=12)	*Acinetobacter spp.* (n=2)	*E. coli* (n=2)	*Streptococcus spp.* (n=24)	*S. aureus* (n=15)	*E. faecalis* (n=10)	*E. faecium* (n=9)	*S. pyogenes* (n=4)
Amikacin	15 (100.0)	13 (100.0)	10 (83.3)	2 (100.0)	2 (100.0)		10 (66.7)	2 (20.0)		
Amoxycillin-clavulanic acid						19 (79.00)				4 (100.00)
Ampicillin	0	0	0	0	0	22 (91.00)	4 (26.7)	8 (80.0)	7 (77.9)	4 (100.00)
Azithromycin						9 (37.5)				4 (100.00)
Cefoperazone- Sulbactam	15 (100.0)	13 (100.0)	10 (83.3)	2 (100.0)	2 (100.0)					
Cefepime	11 (73.0)	6 (46.2)	6 (46.2)		2 (100.0)					
Ceftazidime	10 (66.6)	8 (61.5)	10 (83.3)	2 (100.0)	0					
Ceftriaxone						15 (62.5)				2 (50.00)
Cefuroxime						3 (12.5)				
Cephalexin	0	8 (61.5)	0	0	0	18 (75.00)	7 (46.7)	0	0	2 (50.00)
Cefotaxime	10 (66.6)	10 (76.9)	0	0	0	24 (100.00)	13 (86.7)	0	0	2 (50.00)
Ciprofloxacin	15 (100.0)	8 (61.5)	10 (83.3)	2 (100.0)	2 (100.0)	11 (45.9)	5 (33.3)	8 (80.0)	5 (55.6)	
Clindamycin	11 (64.7)					2 (8.4)	8 (53.3)			
Cloxacillin						2 (8.4)	13 (86.7)			
Erythromycin						18 (75.00)	6 (40.0)	6 (60.0)	5 (55.6)	4 (100.00)
Gentamicin						0	11 (73.3)	8 (80.0)	7 (77.8)	
Levofloxacin		6 (46.2)	6 (46.2)	2 (100.0)	2 (100.0)	9 (37.5)				4 (100.00)
Penicillin						2 (8.4)				
Piperacillin-Tazobactam	15 (88.2)	10 (76.9)	10 (83.3)	2 (100.0)	2 (100.0)					
Tobramycin	11 (73.0)	9 (69.2)	4 (30.8)	2 (100.0)	2 (100.0)					

**Table 4:** Antibiotic sensitivity pattern in bacteria isolated from adenoid core culture. Data presented as n (%) for pathogen sensitivity to a specific antibiotic

	*K. pneumoniae* (n=13)	*P. aeruginosa* (n=12)	*E. cloacae* (n=2)	*Streptococcus spp.* (n=24)	*S. aureus* (n=15)	*E. faecalis* (n=10)	*E. faecium* (n=9)	Coagulase negative *Staphylococcus* (n=4)
Amikacin	9 (69.22)	6 (50)						
Amoxycillin-clavulanic acid				8 (33.33)				
Ampicillin	0	0	2 (100.00)	11 (45.1)	2 (13.3)	6 (60.00)	7 (77.8)	2 (50.00)
Azithromycin				3 (12.5)				
Cefoperazone- Sulbactam	7 (53.00)	6 (50.00)						
Cefepime	6 (46.00)	2 (16.1)						
Ceftazidime	4 (30)	6 (50)						
Ceftriaxone				6 (25.00)				
Cephalexin	4 (30.00)	0	0	7 (29.2)	5 (33.33)	0	0	0
Ceftaxime	4 (30.00)	0	0	11 (45.83)	9 (60.00)	0	0	2 (50.00)
Ciprofloxacin	6 (46.00)	6 (50.00)	2 (100.00)	3 (12.5)	7 (66.7)	4 (40.00)	5 (55.1)	2 (50.00)
Clindamycin					2 (13.3)			2 (50.00)
Cloxacillin					9 (60.00)			2 (50.00)
Erythromycin			2 (100.00)	7 (29.2)	4 (26.7)	2 (20.00)	3 (33.4)	2 (50.00)
Gentamicin			2 (100.00)	0	9 (60.00)	4 (40.00)	7 (77.8)	2 (50.00)
Levofloxacin	6 (46)	2 (16.1)		6 (25.00)				
Linezolid			2 (100.00)			2 (20.00)		2 (50.00)
Penicillin				2 (8.4)				
Piperacillin-Tazobactam	7 (53.00)	6 (50.00)						
Tobramycin	6 (46)	2 (16.1)						
Vancomycin			2 (100)			2 (20.00)		2 (50)

## Discussion

In this study, we observed that besides single organisms isolated in culture, polymicrobial growth is uncommon in both adenoid and tonsil cultures. Though the observed frequency of polymicrobial culture is lower compared to single organisms, there may be a greater likelihood to find polymicrobial infections. Compared to our results, a study from Iran observed multiple microorganisms in 79.6% of cultures from the depth of tonsils [14]. A study from North India also reported a single pathogen being more common in tonsillar core cultures [15]. We observed that the prevalence of Gram-positive bacteria was substantially higher in both tonsil and adenoid cultures. *Streptococci* were the most common bacteria isolated in both cultures. The bacterial profile of the tonsillar core surface varied widely concerning Gram-negative isolates. DeDio et al. reported a common pathogen in tonsil and adenoid tissue in nearly three-fourths of children [16]. Compared to our findings, Buname et al. from Tanzania did not observe any Gram-negative growth in tonsil cultures [17]. There may be a difference in pathogen distribution in different geographical locations. In an Indian setting, a study from Rajeshwary et al. reported the isolation of Gram-negative organisms in adenoids such as *Pseudomonas*, *Klebsiella*, *Escherichia coli*, *Moraxella*, and *Haemophilus* influenza. But, the frequency of Gram-negative isolates was substantially lower than Gram-positive isolates [12]. These results are similar to our observation and indicate that mixed isolates with polymicrobial infection can occur in recurrent adeno-tonsillitis cases. Identifying the microbial profile is essential to plan the strategies. Additionally, differences in the microbiome may be the factor responsible for the development of complications such as otitis media [18].

The majority of pathogens from tonsil culture showed sensitivity to b-lactam antibiotics, fluoroquinolones, and third-generation cephalosporins. A greater degree of resistance was noted in erythromycin and ampicillin. A similar picture was identified in adenoid culture as well. In adenoid cultures, Rajeshwary et al. reported that none of the isolates of *S. aureus* and *Enterococcus* were sensitive to penicillin and ampicillin. They also reported good sensitivity in amoxicillin and clavulanic acid combination and ciprofloxacin [12]. We observed ampicillin resistance in *S. aureus*. Buname et al. reported that *S. pyogenes* and *S. pneumoniae* isolated from the tonsil core were 100% sensitive to penicillin and vancomycin. Methicillin-resistant *Staphylococcus aureus* (MRSA) was reported in 39% of isolates [17]. The presence of such resistant pathogens may be the reason for treatment failures. Taylan and colleagues reported a close relationship between the bacteriology of the tonsil and adenoid flora. They also observed *Staphylococcus aureus* and other beta-lactamase-producing bacteria may be responsible for treatment failures in patients with tonsillitis [19]. Identifying the deep tonsillar bacterial morphology holds the potential for treating the patients accurately as surface, and deep isolates may vary [20]. We observed a substantial difference in the sensitivity of pathogens identified from the adenoid core culture. Thus, our study highlights important differences in pathogens isolated adenoid and tonsil core cultures. This has implications for choosing the right antibiotics in recurrent AT cases.

Our study has certain limitations. Being a single-center study, it would not represent the wide geographical area, and thus results may not apply to other regions of India. We did not assess the clinical symptoms or complications that were associated with recurrent AT; and neither evaluated the impact of surgery on these symptoms. We also did not analyze the patient outcomes post-surgery who would have received culture-guided antibiotic therapy. Also, a comparison of pathogens identified across different age groups and other parameters can provide more insights into the microbial profile of recurrent AT cases.

## Conclusions

Our study highlights that polymicrobial infections are not uncommon in children with recurrent adenotonsillitis. Recurrent AT is associated with polymicrobial infection, and anaerobes can be found with increasing frequency. Though Gram-positive organisms are common, Gram-negative are commonly isolated from tonsil core cultures. We observed that b-lactam antibiotics, fluoroquinolones, and third-generation cephalosporins showed better sensitivity against most of the isolates. Resistance was noted with erythromycin and ampicillin treatment and was the reason for the failure of treatment in many cases. In our observation, culture from the tonsil core and adenoid in children with recurrent tonsillitis and adenitis may not be significantly different in terms of Gram-positive isolates, but Gram-negative organisms may be common in the tonsil core, causing recurrent disease in children. Differences in sensitivities among the pathogens from adenoid and tonsil core cultures might underlie the recurrences of AT despite antibiotic treatment. Thus, understanding the local pathogen sensitivity patterns is essential in recurrent AT.
